# Linking communities to formal health care providers through village health teams in rural Uganda: lessons from linking social capital

**DOI:** 10.1186/s12960-016-0177-9

**Published:** 2017-01-11

**Authors:** Laban Kashaija Musinguzi, Emmanueil Benon Turinawe, Jude T. Rwemisisi, Daniel H. de Vries, David K. Mafigiri, Denis Muhangi, Marije de Groot, Achilles Katamba, Robert Pool

**Affiliations:** 1 Department of Social Work and Social Administration, Makerere University, P.O. Box 7062, Kampala, Uganda; 2 Amsterdam Institute of Social Science Research, AISSR, University of Amsterdam, Nieuwe Achtergracht 166, 1018 WV Amsterdam, Netherlands; 3 College of Health Sciences, Makerere University, P.O. Box 7072, Kampala, Uganda

**Keywords:** Village health teams, Community health workers, Linking social capital, Uganda

## Abstract

**Background:**

Community-based programmes, particularly community health workers (CHWs), have been portrayed as a cost-effective alternative to the shortage of health workers in low-income countries. Usually, literature emphasises how easily CHWs link and connect communities to formal health care services. There is little evidence in Uganda to support or dispute such claims. Drawing from linking social capital framework, this paper examines the claim that village health teams (VHTs), as an example of CHWs, link and connect communities with formal health care services.

**Methods:**

Data were collected through ethnographic fieldwork undertaken as part of a larger research program in Luwero District, Uganda, between 2012 and 2014. The main methods of data collection were participant observation in events organised by VHTs. In addition, a total of 91 in-depth interviews and 42 focus group discussions (FGD) were conducted with adult community members as part of the larger project. After preliminary analysis of the data, we conducted an additional six in-depth interviews and three FGD with VHTs and four FGD with community members on the role of VHTs. Key informant interviews were conducted with local government staff, health workers, local leaders, and NGO staff with health programs in Luwero. Thematic analysis was used during data analysis.

**Results:**

The ability of VHTs to link communities with formal health care was affected by the stakeholders’ perception of their roles. Community members perceive VHTs as working for and under instructions of “others”, which makes them powerless in the formal health care system. One of the challenges associated with VHTs’ linking roles is support from the government and formal health care providers. Formal health care providers perceived VHTs as interested in special recognition for their services yet they are not “experts”. For some health workers, the introduction of VHTs is seen as a ploy by the government to control people and hide its inability to provide health services. Having received training and initial support from an NGO, VHTs suffered transition failure from NGO to the formal public health care structure. As a result, VHTs are entangled in power relations that affect their role of linking community members with formal health care services. We also found that factors such as lack of money for treatment, poor transport networks, the attitudes of health workers and the existence of multiple health care systems, all factors that hinder access to formal health care, cannot be addressed by the VHTs.

**Conclusions:**

As linking social capital framework shows, for VHTs to effectively act as links between the community and formal health care and harness the resources that exist in institutions beyond the community, it is important to take into account the power relationships embedded in vertical relationships and forge a partnership between public health providers and the communities they serve. This will ensure strengthened partnerships and the improved capacity of local people to leverage resources embedded in vertical power networks.

## Background

Linking people with existing health care services is a role of any effective and efficient health care delivery system. Low-income countries like Uganda that are faced with shortages of health care resources [1, 2], community-based approaches, and in particular involvement of less-specialised cadres in health promotion have been advocated in global health discussions as means of linking people with existing health care resources [3, 4]. Following the 1978 Alma-Ata Declaration, community health workers (CHWs) gained centre stage in “accelerating coverage of essential interventions particularly for the poor and underserved communities” [5]. The adoption of CHWs is partly due to suggestions that they increase coverage and cost-effectiveness of health services delivery [6], provide an alternative solution to the crisis of health workers in resource-constrained communities [7] and a basis for people participation in community health [8].

**Fig. 1:**
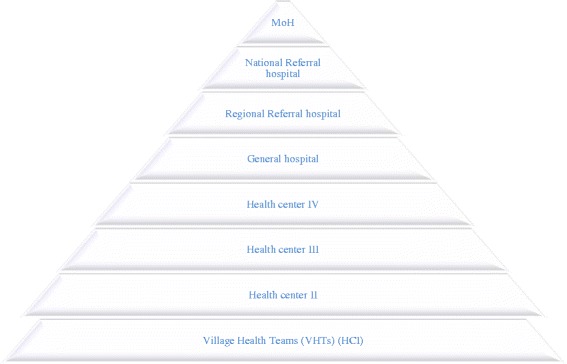
Uganda’s health system structure placing VHTs at the bottom of the hierarchy [65]

Village health teams (VHTs) fall broadly within the CHWs model. VHTs were introduced in Uganda in 2001 as part of the implementation strategy for the 2001–2006 national health strategic plan; they fall within the broader government’s health sector strategic framework which emphasizes “the client and community” [9]. The focus of VHTs as a component of health promotion is the “more active and meaningful participation in health development…” of communities [9]*.* Contrary to the WHO recommendations of a “hub and spoke” model of primary health care that places community at the centre of a health system [10], in Uganda, decentralized national health care delivery system follows a tiered structure with VHTs occupying the lowest level. At the top of the structure are the national referral hospitals followed by regional referral hospitals, general hospitals and health centre (HC) IVs, HCIIIs and HCIIs; VHTs (HCIs) occupy the bottom of the ladder [9] (see Fig. 1).

The 2010 national VHT strategy that operationalises and institutionalises the VHTs in health services delivery is particularly emphatic about their linking role. In addition to performing major primary health care roles, the Ugandan national VHT strategy projects VHTs as “the first link between the community and formal health providers” serving to “link the communities to the formal health service delivery system”[11]. The national VHT strategy further states, “VHTs will help bridge the gap that exists between un-served households and the formal health system” and that VHTs will be people’s “first contact with the health system” [11].

The strategy of VHTs linking communities to formal health care is based on the understanding that access to formal health care remains a challenge for Uganda’s health care system. For example, whereas over 72% of the population lives within a recommended 5-km radius of a health facility, only 44.4% of expectant mothers deliver at health facilities and only 32.4% of pregnant women attend all four recommended antenatal visits [12]. Factors that affect accessibility emanate from both demand and supply sides of health service delivery system [12–15].

Unlike in countries like South Africa where community health workers are based at health facilities and directly answerable to the health service that selects them [16], in Uganda, the VHTs took on a normative view of CHWs as community-based generalists, selected by the people in the community, but they report to the structures of government, mainly formal health care service professionals.

Successful examples of CHWs include Brazil’s family health program [17] and others in Bangladesh and Nepal [18]. Studies elsewhere equally demonstrate that CHWs have positive effects on health because they act as “entry points”, “links”, “bridges” or “connectors” between communities and formal health care services, systems and resources [4, 5, 18–21]. However, some reviews that counter such claims suggest that CHWs “have suffered from poor integration into the broader health system” and that they are not a solution to weak health systems [22]. Such reviews have tended to base their evidence on factors such as high levels of attrition, lack of motivation and poor incentives for CHWs [23–25].

Whereas there is consensus that CHWs, due to their close ties to communities, are best placed to link communities with formal health care providers, there is hardly any empirical evidence in Uganda to support or dispute such claims or show how these linkages should materialise. Haines et al. point out that one of the main challenges facing effective utilisation of CHWs is unresolved concerns about CHWs as either an extension of the authoritarian formal health care system into the community or CHWs as agents of community change [5]. Therefore, using linking social capital framework, the objective of this study is to examine and challenge the claim that VHTs create “linkages”, “connections” and “bridges” between communities and formal health care providers. Specifically, we seek to answer the following questions: what explains the failure of VHTs to link communities to formal health care? Second, can linking social capital help explain the failure of VHTs to link communities to formal health care providers? This paper builds on an earlier paper that explored the VHT selection process and performance and particularly the best way to draw volunteers from a community, without relying on financial incentives [26]. In this study, however, we go beyond the selection process and argue that besides the VHTs selection going wrong, the failures of the VHTs in linking community members to formal health care can be explained by examining issues about the transition failure, the power relations among health care system stakeholders, the position of the VHTs as an extension of a formal health care system and the community lack of trust in the formal health care facilities. Our analysis of the networks and systems involved is informed by community members’ perception of the roles of VHTs, the formal health workers’ perceptions of VHTs and the VHTs’ perception of their own position in a rural community in Luwero District, Uganda. In-depth insight into the way VHTs form a link between communities and formal health care providers contributes to discussions on the effectiveness of CHWs in promoting community health in low-income countries and particularly their potential role as an alternative to the human resource deficiencies in the health sector.

### Linking social capital and health care access: a theoretical framework

Defined as a social resource accessed through a person’s networks and participation in community events, the concept of social capital became a focus of health research following calls to move away from the traditional curative health care services towards contextual and social determinants of health [27–30]. Social capital theorists and empirical studies project a positive link between social capital and a community’s health outcomes because it creates bonds within communities, connects communities with each other and links them to formal health systems [29]. Ogden et al. further suggest that various forms of social capital—bonding, bridging and linking—are critical to strengthening health systems. Bonding social capital relates to exclusive solidarity among people who are alike while bridging social capital relates to inclusive solidarity between people from varying backgrounds [31]. Features of social capital have also been used to explain enrolment into community-based health insurance schemes (CBHI) in Senegal [32].

Linking social capital, the focus of this paper, involves the accumulation of ties with individuals in power and institutions of influence usually outside the community [31]. Linking social capital constitutes “resources found in vertical relationships…between individuals in a community and institutions or individuals with access to resources beyond the community” ([33], p.283). In this case, formal health care facilities constitute “resources beyond the community” [33] and VHTs “conduits for feedback from the community” ([29], p.4) acting as a vessel for leveraging these resources. Linking social capital is a useful framework; it serves as a lens for understanding how claims of linkages between communities and formal health care providers materialise or do not materialise under the influence of VHTs.

Linking social capital is based on key assumptions about the perceived linkages. First, linking social capital assumes that communities will have “access to networks or groups with relatively more power and decision-making influence” ([29], p.1078). This suggests that the starting point is to assess the “networks that connect people across explicit vertical power differentials” ([31], p.2205). Second, linking social capital is premised on the assumption that people have the capacity to engage vertical connections to obtain resources from formal institutions such as health care providers [31]. Given that health inequities in populations continue to be contingent on a lack of access to equal distribution of power [34, 35], any intervention such as VHTs that seeks to provide a way of engaging power structures and access to such resources is critical. Third, linking social capital assumes that from engagement, a “partnerships between health care providers or public health entities and underserved communities” is formed that makes extending of primary health care services to the vulnerable communities possible ([33], p.278). Partnerships also imply “a systems model of collaboration” in which different players support each other to achieve common goals [29]. Within this partnership, there are also echelons of power, particularly the power of the professional health workers or even the power that the VHTs themselves assume after becoming VHTs in their community. Power and influence are therefore central to the discussions of linking social capital. Where power and influence are concerned, social inequalities arise. In fact, one of the earliest notable concerns of the concept of social capital was based on the premise that it reproduces social inequalities in society [36]. Evidence shows that interventions originating from outside the community create inequalities and asymmetries of power in communities [37, 38], affect local innovations in managing community problems, increase dependence on external actors [39] and potentially facilitate elite capture [40, 41].

Some studies have shown that linking social capital can in itself be deleterious if it is not based on the existing bonding and bridging forms of capital in the community [31]. Others have argued that social capital in form of bonding social capital has an impact on how structures such as VHT function [42]. Indeed, community-based interventions like VHTs are spearheaded on the assumption that the actors know communities better and can effectively utilise existing social bonds not only to mobilize but also link community members to formal health care. Studies have shown that CHWs, given their close links with local communities, are well placed to link vulnerable populations to the available health resources [5], which in itself suggests that they are a resource and base their power to promote health on their links with communities.

Despite the evidence suggesting a positive relationship between social capital and health, the application of social capital as a measure of understanding health care access has been growing rather slowly. A systematic review of 2396 abstracts by Derose and Varda found only 21 papers with “some measure of social capital and its effects on health care access” [33]. Derose and Varda show that most of the research has tended to focus on the health outcomes particularly health care utilisation with little attention to access. Therefore, linking social capital remains the least developed in health research [33]. By applying linking social capital framework in this study, we hope to add to the development of the concept and highlight its usefulness in explaining the successes and failures of community-based interventions like VHTs affect health care access for vulnerable populations.

### VHTs in Luwero District

The 2010 Uganda national VHT strategy specifies that VHTs should be selected by and from within the community. The recruitment of VHTs was carried out as a measure to harmonise and streamline earlier community-based tasks such as community-based growth monitors, community-owned resource persons, community drug distributors, home care providers, counselling aides or parish mobilisers among others [11]. In fact, most of the recruited VHTs had one or more of the above responsibilities.

In Luwero District, recruitment and training of VHTs was undertaken around 2006 by an NGO, the Africa Medical Research Fund (AMREF), under what was then known as the Malaria, HIV and AIDS, and Tuberculosis (MART) project. After recruitment, the VHTs received training before they started working as VHTs. The training was meant to equip them with basic knowledge, skills and attitudes necessary for understanding and helping communities with their health concerns. Upon selection, each VHT would be assigned at least 25–30 households implying that the number of households in a village would determine the number of VHTs to be selected per village. In the community where fieldwork was done, a total of five VHTs were selected per village. When the MART project ended, the support of VHTs was left to the local government [26].

Studies have looked at how the VHTs mobilise communities for health programmes that come into the community such as immunisation and outreach activities [43, 44]. In some areas like post-conflict northern Uganda, several NGOs have implemented activities that directly provide incentives for the VHTs and health workers to perform their duties [44]. As a result, VHTs in such communities have been able to perform their duties such as home visits, community mobilisation, management of common ill health conditions and drug distribution, follow-up of expectant mothers and post-natal mothers, distribution of health commodities and malarial control [44]. Although Kimbugwe et al. found that VHTs are critical agents for bridging the gap among the community, health facilities and other key development partners in the health sector, an earlier study conducted in Yumbe in northern Uganda posits that the achievements registered by VHTs should be considered with caution, arguing that the VHT programme is a delicate venture that depends on the means by which VHTs and those who supervise them are motivated [43]. Studies conducted in mid-western and south-western Uganda on VHT motivation and retention concluded that VHT motivation is critical for their performance [23, 25]. In our study of VHTs in Luwero, we found that the selection process of VHTs was fraught with distrust, which at times seemed to have affected their legitimacy [26]. However, studying the selection and motivational processes gives an incomplete picture of the failures of VHTs. We argue that a complete picture lies in examining the nature of interactions among the actors within the health systems. Almost all previous studies on VHTs in Uganda, save for Turinawe et al., have been largely cross-sectional studies which provide little information about the experiences of the VHTs in providing the much needed linkage between the community and health service providers. Uganda is politically, economically and socially diverse with multiple socio-economic and political realities. Therefore, these multiple contexts impact, in a number of ways, how VHTs perform their duties. To our knowledge, there is no study using ethnographic approaches that has examined the claim that VHTs serve to link communities to formal health care providers.

## Methods

This paper is based on ethnographic fieldwork conducted between 2012 and 2014 in rural Luwero, Uganda. Luwero District is located approximately 60 km from Kampala, the country’s capital. The recently concluded national housing and population census indicates that Luwero district has a total population of 456,958 persons [45].

The community where fieldwork was conducted is relatively remote, located approximately 5 km off the Kampala-Gulu road, north of Kampala city. The term “community” is used here to refer to a collection of villages from which the participants were drawn. The prominent feature in this community is a trading centre called Dekabusa. With several small-scale businesses, retail shops, saloons, local clinics, makeshift video halls, bars and *chapatti* selling stalls, Dekabusa is an important centre of this community. Most adults in this area own a mobile phone and a radio, which keep them informed and in touch with friends and relatives outside the community. The main means of transport, motorcycle taxis (*boda bodas)* have also become a lucrative business for young men from this community and help to keep the community linked to the facilities outside the community such as markets and health facilities.

The majority of the residents described themselves as small-holder peasant agricultural farmers; however, some of them still had small-scale businesses to supplement their incomes. Although a proportion of the people regarded themselves as native Baganda, due to intermarriages, the community is more ethnically diverse. The native Baganda and non-Baganda speak Luganda, the local language through which the interviews and discussions were conducted.

### Data collection

The first three authors conducted ethnographic fieldwork between 2012 and 2014 as part of a bigger project, “Developing Sustainable Community Health Resources in Uganda” (CoHeRe). The ethnographic data for this paper were collected mainly by the first author and results are presented in the first person when they derive directly from his fieldwork experience. The main method was participant observation. The first author joined a village savings and loan association (VSLA), called *Bajja Basaga—*with a membership of 22 members—participated regularly in the weekly savings meetings, from January to December 2013. As a member of the VSLA, the first author saved money weekly, documented members’ interactions and type of information shared during meetings, how meetings were conducted and what VSLA members and non-members talked about with regard to health issues in their everyday interactions. Some of the VSLA members were also VHTs themselves. Participation in the VSLA allowed me to gain full entry in the community and consequently got access to how community events such as burials were organised. VSLAs have a social fund called ebigwa bitalaze (unforeseen circumstances) where members save money to help members during crises such as death and sickness. Whenever a person died in the community, VSLA members were often part of the team involved in digging the grave, fetching water and other communal arrangements. It was in such spaces that a number of issues around health were talked about and understood. During burials, people often discussed issues about the deceased’s cause of death, which often rotated around his/her health. For example, when one of the community members (Okello) lost his son, people talked about his health condition during burial arrangements including how one of the VHTs had engaged the family about the boy’s health condition.

Although participant observation was a continuous engagement, general observation points and activities involved spontaneous interactions with people in bars, playing ludo (a board game) with youth, attending church and religious functions, contributing money whenever a member of a community was sick, participating in burial activities, playing with children and attending community meetings and health outreach programmes. As Spradley notes, our participation was guided by activities and the physical aspects of the situations “as and when they unfold[ed] in their natural settings” ([46], p.54). Focused observations were made in a total of seven village meetings, two health outreach campaigns organised through VHTs, four burial ceremonies, three wedding ceremonies, one church fundraising function and several meetings in VSLAs. Field notes written after every day’s work were used as points of reflection among the CoHeRe team members and follow-up in subsequent observations, interviews and focus group discussions (FGDs).

In addition, a total of 91 in-depth interviews (36 with males and 55 with females) and 42 FGDs (23 with females only, 15 with males only, four mixed FGD) were conducted with adult community members aged 18 and above. Given that this study was conducted as part of a broader CoHeRe project, initial interviews and FGDs focused on broad issues including community groups, forms of togetherness, health problems and the roles of VHTs in addressing some health problems. After preliminary analysis of the data, and identifying the role of VHTs, we conducted extra six in-depth interviews and three FGDs with VHTs and four FGDs with community members on the perceived roles of VHTs making an overall total of 97 interviews and 49 FGDs. All interviews lasted between 30 min and 1 h and were recorded with permission from the participants. In-depth interview participants were selected using a simple random procedure while VHT participants were purposively selected. Except for the FGDs with VHTs, which depended on how many VHTs were available, FGDs with community members usually involved between 10 and 12 participants and also lasted 30 min to over an hour.

In addition, seven pile sorting sessions were conducted with groups of women, men and youths to understand how lay community members evaluate the community health problems and interventions [47]. During these sessions, participants of between 6 and 12 people responded to three main discussion points. First, participants identified the main health problems in the community. Second, using a total of 100 beans each of the identified community health problems were ranked in their order of severity. Problems considered most severe were allocated most beans. Third, after ranking the health problems, participants discussed existing interventions/solutions including the role of VHTs in addressing each of the ranked health problems. Key informant interviews were also conducted with representatives from the local government, health workers at the existing public health facilities and district health department and NGOs operating in the area.

We also reviewed Uganda government 2010 VHT strategy, policy and reports on VHTs.

### Data analysis

All community interviews and FGDs were conducted in Luganda, audio recorded and thereafter transcribed and translated into English by the first author. The transcription process was highly iterative and involved reflecting on the data, discussing emerging issues with CoHeRe team members and following up the data in subsequent interviews. For example, after preliminary analysis of 91 interviews and 42 FGDs from the bigger CoHeRe project, the role of VHTs emerged as one of the themes. We then conducted an extra six in-depth interviews and three FGDs with VHTs and four FGDs with community members on specific questions about the perceived success/failure of VHTs, making an overall total of 97 interviews and 49 FGDs from which analysis for this paper was done. However, the knowledge gained about the structures in the community derived from the entire CoHeRe project was equally critical in informing our general interpretation and analysis of the results presented in this paper.

Data was inductively coded using Nvivo10 software by the first author. Codes were discussed with CoHeRe team not only to achieve concordance but also as a basis for refining codes [48]. Whenever a text was coded, we would reread the text and thoroughly search the entire data set to explore any competing explanations. This process allowed new codes as child nodes to be added whenever relevant. The text codes were then developed as themes emerging from analysis. These themes include enthusiasm about VHTs, perceptions that VHTs work for “other” people, VHTs as a tool to control people, masking the challenges of the health care system, government neglect, lack of support and recognition from formal health care providers and perceived failure of VHTs to address social determinants of health.

In addition, analysis of government policy documents, VHT strategy and reports on VHTs was conducted for their content and relevance to either illustrating or questioning the narratives from interviews and FGDs.

## Results

Results in this section are arranged based on the key themes from the data: enthusiasm about VHTs, perceptions that VHTs work for “other” people, masking the challenges of the health care system, VHTs as a tool to control people, government neglect, lack of support and recognition from formal health care providers and the perceived failure of VHTs to address social determinants of health in the face of an ailing health care system.

### From early enthusiasm to sceptical apathy

The recruitment and training of VHTs in Luwero District in 2006, according to discussions with community members and VHTs, generated enthusiasm among the local people and the VHTs, especially when the first incentive-led rewards for VHTs yielded creditable results as VHTs interacted with community members and formal health care workers. AMREF, the NGO that trained VHTs, initially provided financial incentives and facilitated their work; for example, they routinely wrote reports and submitted them to their supervisors. They were given bicycles, T-shirts and metallic boxes for keeping drugs they were meant to distribute.

For the community members, the VHT strategy created a belief that VHTs were there to solve all their health problems. These expectations were heightened by the initial enthusiasm with which the VHTs performed their duties under supervision from the health workers. As a result, the interactions between the VHTs, the health workers and the community members showed signs of hope. The community members believed in the VHTs’ work and took their support seriously.

One day as I moved around mobilizing people for vaccination, I came across a home that I had never visited. When I arrived, I saw this child lying down. His mother told me he was six years old. This child was six years but could not move at all; he was lame and I think the parents did not even know what to do with the child…So, I gave them a form and we filled it out together. I told them to take the child to a rehabilitation centre called Katalemwa. They took the child there, the doctors helped him with walking aids and with time, he gained strength and he is now doing well….This was about three years ago. (Interview with a VHT)

Nonetheless, the early bout of enthusiasm gradually waned into indifference and feelings of desperation when community expectations of receiving drugs from VHTs were not forthcoming.

Ever since they (VHTs) were trained up to now, they (VHTs) have never received any Coartem (anti-malarial) to distribute. Yet they (VHTs) were given T-shirts and boxes where they were supposed to keep medicines but up to now, they only have empty boxes and won out bicycles. (Interview with Health Assistant)

According to the community members, the failure to equip VHTs with essential medicines to distribute to community members whenever sick resulted into a loss of trust of the VHTs.

Respondent 1: I wonder why they said those people [VHTs] were going to be our village doctors. Even witchdoctors have what they need to use. Now for VHTs, they are here; we see them every day, but they have nothing (no materials) to help us with. (FGD with community members)

This statement suggests that community members had expectations of the VHTs, which were not met.

### VHTs work for “other” people

In one FGD we conducted with community members, participants unanimously mentioned “They (VHTs) work on instructions from their bosses. They do what they are told to do.”

The label “bosses” was echoed in most discussions with community members to refer to the officials at the district health office, district hospital and NGOs who use the VHTs to undertake community mobilisation during health campaigns, immunisation programs and other outreach activities. The district health office keeps phone contacts of all VHTs from which they are called to mobilise communities. Take an example of a community health and personal hygiene sensitization outreach event organised on June 18, 2013, by the district health department, supported by Korean International Cooperation Agency (KOICA) and Sahmyook University based in Korea. The community members who attended (11 male and 15 females) were mobilised and informed about the program by the local council (LCI) chairperson who was a member of the VHT. He had been given instructions to mobilise people on phone two days to the event.

During a discussion with the in-charge (the head of the health workers) at Kasana HCIV, he revealed that VHTs were critical mobilisation agents who are occasionally given an activity allowance to mobilise communities for government programs like distribution of insecticide-treated mosquito nets.

Whenever there is an outreach the VHTs are the people we use to mobilise for us. Recently we used VHTs to distribute insecticide treated mosquito nets.

Besides the mobilisation of community members on behalf of the formal health care providers, sometimes VHTs are trained to implement programs initiated by NGOs or government in the community. Community members interpreted such actions (using VHTs to mobilise communities and training VHTs to implement NGO or government programs) to mean that health workers were using the VHTs to pursue their (health workers) interests. In an interview with a health assistant, she recounted how the community members tasked her during one of the health outreaches to explain why the VHTs only work for the health workers.

Fears that the VHTs were working for the “other” were heightened by accusations that they were drawing salaries and other favours from the government; this is likely attributed to the earlier financial incentive-led work they did in the community and the fact that VHTs are given an allowance whenever engaged in a short term activity by the “bosses”.

Respondent 7: If you go out to advise someone to go to the hospital, he or she will tell you, “you are telling me, yet you are the ones who receive salaries, you get some of the money you are paid so that you take me to the health facility; you are government workers”. They take us as government workers; they think we get salaries. (FGD with VHTs)

We also learnt that local government authorities sometimes used police to enforce hygiene standards at community level.

VHTs from one of the parishes came and told me that “we are tired of people in our parish who do not want to construct latrines. You should come with police for us we are ready to take you to every home that has no latrine and arrest them”. With the help of VHTs and the LC chairpersons, we got police and a car from the district and arrested the people who had no latrines and charged them (Interview with health assistant)

While reflecting on the use of VHTs as police agents to arrest people, some community members wondered why the same was not done to VHTs who did not have latrines and were not showing a good example. While we did not witness any arrests of such nature by VHTs or police during fieldwork, VHTs told us that they had to stop because it was leading to hatred.

We learnt, however, that what the community members perceived as working on the instruction of “others” is a function enshrined in the VHT national strategy. It states: “All health activities at community level by the government, NGOs and or partners targeting communities shall be coordinated through VHTs.” ([11], p. 19). The assumption was that by implementing all activities through VHTs it would make “the community value and continue demanding and utilizing their services” ([11] p.19). However, discussions with community members depict VHTs as powerless; they do what “their bosses” want them to do than what the community needs. To some of the community members, the VHTs were simply a programme like any other programme that comes and goes. The participants positioned the VHTs as a shop that was opened, did not serve its purpose and should be closed. However, some community members blamed their (VHTs) failures on the government’s inability and perceived lack of interest in equipping VHTs with the necessary facilities including medicines to distribute to the people.

### VHTs as a tool to mask the challenges of the health care system

Facility-based health workers mentioned that VHTs are not “experts”, yet they seem to expect preferential treatment.

They [VHTs] even want to bring patients and skip the queues! It is not possible, those people are not experts; they should simply refer people here. If they send someone to our centre, he/she should come as any other regular patients. (Interview with district health official)

The VHTs we talked to noted that the patients they refer should not be made to line up at the health facilities, which, to them (VHTs), is a source of motivation for people to visit health facilities. These conflicting expectations reveal some tensions and minimal contact between the formal health care workers and VHTs.

Some health workers also questioned the idea of introducing VHTs and wondered why the government that failed to equip the health centres with drugs, equipment and medicines would do the same for VHTs at the community level and later on be able to motivate them (VHTs) to link the community to formal health care facilities. A health worker wondered, “If the health facilities are not equipped with drugs, how do the VHTs refer people here?”. While public health facilities offer free medical services including drugs, government reports have often reported drugs stock outs [49]. VHTs told us it was fruitless to refer people to health facilities that are ailing with no drugs and equipment.

Respondent 7: There are those (people) that have lost hope in the health facilities; whenever they go there they do not get medication.

Respondent 3: When you ask someone to go to the health facility, they will ask you if you are sure that they will get drugs. You are not even sure what to tell them; in the end, people stay home. (FGD with VHTs)

The facility-based health workers also mentioned that public health facilities are not well equipped.

Here [Kasana HCIV], we do not have an operating theatre. How can that be? The doctor is here, everything, and you just cannot put up a theatre? … I think the government was not honest in saying that they wanted to give anti-malaria drugs to the VHTs. How can they do that if the hospitals are not stocked with medicines? (Interview with the person in-charge at Kasana HCIV)

According to the Ministry of Health structure, HCIVs are supposed to have an operating theatre. The revelation by the person in-charge that the HCIV that serves the study community lacked an operating theatre was in itself revealing of the bigger challenges facing the formal health care system. According to the health workers, the creation of VHTs demonstrates the government hypocrisy and an attempt to mask the health sector challenges. The arguments made by health workers were that the government could have simply facilitated the health inspectors and health assistants who are already in the national health care delivery structure, instead of poorly facilitated VHTs to do their work.

The health workers argued that after failing to equip the health facilities and structures such as health inspectors and health assistants, the government recruited the VHTs to work as volunteers. When we talked to the VHTs, they mentioned that AMREF initially facilitated them with a monthly allowance to do their (VHTs) work, and some VHTs received other incentives like T-shirts, bicycles, among others. The monthly facilitation allowance stopped when the VHTs were transferred to the local government structure because it was too expensive for government to manage. At the time of fieldwork, the VHTs only wait to do work occasionally given to them by NGOs working in the area (e.g. the mobilisation of community for KOICA community hygiene campaign) and special government programs like immunisation or distribution of insecticide-treated mosquito nets. When they do this work, they (VHTs) receive a facilitation allowance. The idea that VHTs were supposed to do the work voluntarily was, according to the health workers, an attempt to shift responsibility.

If we were to go by this argument, it would also imply that the fears of health workers are embedded in the realisation that by fronting the VHT structure, the government was inhibiting the effective functioning of formal health systems. In fact if the government mooted idea of creating “the health extension workers” (HEWs) [50, 51] goes through it would then completely sidestep the positions of health assistants and health inspectors and continue to mask critical health sector challenges.

### VHTs as a tool to control people

The creation of the VHT structure, according to some health workers, was simply a ploy by the government to control people after failing to meet the cardinal obligations such as equipping health facilities. In some discussions, health workers argued that the government established such structures as VHTs to facilitate the “politics of control” over people and use them (VHTs) to mobilise support for the ruling regime during elections.

There is something you do not understand about the politics of control. You see, these structures like VHTs and Local Councils and all these groups being formed are to help the government in case of organized rebellion. The problems they set them up to address are just used as an excuse. Politicians use them (VHTs) as mobilizers during elections. (Interview with the in-charge Kasana HCIV)

However, given that when we did fieldwork there were no elections organised, we could not competently verify this claim. What we note, however, is that contrary to the VHT strategy guidelines that spelt out that political leaders, especially local council leaders at village level were not supposed to be elected as members of VHTs, we found that almost all the VHTs in this community were either local council chairpersons or members of the local council executive committee.

### Government neglect, lack of support and recognition from formal health care providers

The public health services delivery structure in Uganda places VHTs at health centre I (HCI) at the community level and they serve as the first point of contact. VHTs revealed their uncertainty about their position in the structure. They mentioned that the government abandoned them by reneging on the promise to give them medicines to distribute in the community. Some VHTs mentioned that they struggle to get attention and recognition from the formal health care providers. In one FGD with VHTs, they mentioned that they are not recognized and the attitude of the health workers leaves them uncertain of their position in their interactions.

Respondent 7: The VHT is not taken as someone who is part of the formal health system. The health workers at the government health facilities do not respect you; so, you find that as a VHT you lose interest in following up issues. For example, we have many sick people but we no longer know them. Making follow ups at the health facilities, for example, TB or even HIV patients is no longer done because our bosses (government and district officials) are no longer helpful. (FGD with VHTs)

The attitudes of formal health care providers, according to VHTs, amount to humiliation that partly contributes to the lack of interest in VHT work.

The truth is that we lost interest in the VHT work, not only in this village, but elsewhere too. Those days (when VHTs had just started) you could call the health assistant to help you, but now they first ask for transport; sometimes they ask you, “Who are you to call me?” (Interview with VHT)

The VHTs’ complaints about the lack of support to follow up on sick people in the community appear to push their work to perfunctory advice on basic health issues. For example, on January 17, 2013, Mr. Okello, a community member, lost his son of 4 years. Before his death, one of the VHTs (John) had advised Okello and his wife to take their son to a bigger hospital in a neighbouring district where they had been referred by the medical doctor at a public health facility for further tuberculosis (TB) tests and treatment. When we spoke to John, he told us the boy was not taken for further TB tests because his parents did not believe it was TB. According to John, the parents believed the boy had been bewitched by one of his relatives. When asked what he thought the problem was, John said that whenever he visited, the boy’s parents always claimed they did not have sufficient funds to take the boy to the hospital, yet “the mother runs a food joint and the father is a businessman.” Surprisingly, according to John, the boy’s parents found money to take the boy to traditional healers three times before his death. In the interpretation of the problem, John said “helping a person who has not asked for help is hard.” The VHTs also mentioned lack of support from the formal health care system to overcome barriers presented by a clash between community cultural understanding of illness and disease and the biomedical interpretation. If a VHT hits a deadlock, i.e. if a community member does not take the VHT’s advice, VHTs have no available support from the formal health care system to help them overcome this challenge.

### Failure to address social determinants of health

The 2010 Uganda National Health Policy identified several social determinants of health including household income, education, status of housing and social and cultural beliefs [14]. The 2006 Uganda Demographic and Health Survey showed a direct relationship between poverty and various health indicators including prevalence of diseases [52]. Poverty, poor transport networks and distance to health facilities limit community members’ access to health care.

In one of the pile sorting sessions, participants argued that poverty and poor hygiene are greater health problems than the lack of health facilities. One person stated, “… you would not worry about treatment facilities if you had money to access them even if they are far away from the community.” The nearest private and public health facilities are approximately 5 km away, and it costs about USh 2500 (Ugandan Shillings, equal to US$ 0.70) to travel this distance by *boda boda*. Participants in pile sorting sessions mentioned that such problems are solved by “mobilising ourselves”, which meant actively marshalling resources including borrowing money from friends and neighbours, organizing transport and caring for “unable” community members. None of the participants mentioned relying on VHTs for solving some of these problems.

For the most part, VHTs acknowledged factors beyond their own control. The VHTs mentioned that even in rare situations when people received their (VHTs) advice to visit a health facility for any ailment, they still needed money and transport to enable access to a health facility.

Respondent 1: Many people fail to access health care because they do not have money, others might have the money but fail to reach the health facility, maybe the *boda bodas* that usually take them are not available.

Respondent 5: Once, I went to someone’s home and found that the woman was at the point of delivering a child. I told her to go to a health centre. She explained that she did not have any money to get there … This showed that our only difficulty was finding a way to get to the health centre. (FGD with VHTs)

The reflections captured in the discussion with VHTs above show that accessing a health facility for rural people requires much more than giving advice and information about the existence of a service at a health facility as is currently done by the VHTs.

## Discussion

Although there is a significant reference in the literature that VHTs and CHWs in general, serve to “link”, “connect” and “bridge” communities with formal health care providers [4, 5, 9, 11, 22–24], empirical evidence on how that should materialise remain scanty. In what they describe as the “determinants of the success of community health worker programs”, Haines et al. identify factors that determine the success of a CHWs programme to include socio-economic and political, health systems, community and international factors ([5], p. 2125). While Haines et al. examine the challenges faced by CHWs in general, we found that explaining the failures of CHWs to link communities to formal health care providers begins by examining the nature of interactions among health care systems stakeholders. Linking social capital framework which explores these interactions and offers an understanding of how a systems model works and “systematic mechanisms for partnerships across power structures” are created offers a starting point ([29], p. 4). The findings point to key critical aspects: transition failure, VHTs as an extension of formal health system, power relations between formal health care workers and VHTs and limitations of an ailing formal health care system.

### VHTs and transition failure

When AMREF recruited VHTs, trained and facilitated their work, VHTs performed to the satisfaction of the community and the health care providers. VHTs received financial incentives from AMREF in the early phases of the programme. People appreciated this period where the VHTs were active, wrote reports and through supportive supervision they were able to regularly interact with the formal health care workers. From the linking social capital perspective, it is evident that AMREF facilitated the VHTs to act as a vessel for leveraging “resources beyond the community” [33]. Linking social capital is premised on the assumption that communities have “access to networks or groups with relatively more power and decision-making influence” ([29], p.1078), which in the early phase of AMREF work with VHTs was attained. However, from AMREF to government structures, there was a *transition failure*, which culminated in “accusations” from community members and VHTs of government neglect, failure to equip the VHTs and sustain an incentive structure initiated by AMREF, which affected the linkages. This finding is supported by Golooba-Mutebi’s discussion of similar experiences with the implementation of health unit management committees (HUMC) in Mukono District, Uganda [53]. Golooba-Mutebi found that HUMC were successful at a time when AMREF, implementing a World Bank project, paid allowances to members of HUMC to attend meetings and write reports but HUMC nearly grounded to a halt as they transitioned to the government formal structures [53]. While linking social capital espouses partnerships as an essential ingredient in the processes that involve connecting people to formal social services [33], this finding suggests that potential partnerships for effective linkages can be seriously hampered if other interested stakeholders become central players at a later stage in an intervention. For example, the local government structure that was meant to be a central player in managing the VHTs including the incentive structure appeared absent in determining the incentives of the VHTs during their selection. In such a case, the success of the program is left to how well the transition is managed, which in this case was poorly managed.

### VHTs as a top-down model

The community interpretation of the work of VHTs as working for and under instructions of the “other” suggests that community members perceived VHTs as an extension of the formal health care system into the community. Whereas this may not necessarily be a bad thing, theoretically, literature on linking social capital posit that external interventions that draw little input from the community tend to generate resentment and corruption and instead of being beneficial, resources from these agencies become deleterious [31]. In other words, while the formal health care providers appear to have gained influence at the community level, there is little community interests and needs permeating through VHTs onto the formal health care system. As Haines et al. observe, such unresolved arguments rotate around perceptions of CHWs as either an extension of the authoritarian formal health care system into the community or CHWs as agents of community change that serve the interest of communities [5]. The VHTs in Luwero appear to be more of the former than the later. The results support earlier evidence that demonstrate how community members interpreted the work of VHTs as policing and saw VHTs as allies of a formal power structure instead of as an alliance with the community members whom they were meant to serve [26].

Community members perceived the VHTs model as a top-down structure that executes instructions which contradicts the argument that a community-based health care programme emanates from the community [4, 27]. Evaluations of CHWs hardly adduce evidence to show that by allying with the formal health care structure, the VHTs alienated their functions in the community, which affected their ability to link communities to formal health care. But at the same time, their perceived alliance with formal health care has not been helpful since the formal structure hardly provides any support to VHTs to do their work as we have seen with Okellos case.

While it often assumed that given their base in the communities, CHWs understand the local needs, health beliefs and customs and are, arguably, better placed to link community members to existing formal health care providers [5], our findings show that the role of VHTs did not often conform to these assumptions. This is partly because VHTs, and by extension CHWs, may not necessarily be representative of the community [26]. Community discussions suggest that although VHTs have close links with community members, the VHT strategy was conceived with little reference to how the initial bonds in the community would facilitate the successes or failures of the programme. This then suggests that being able to link communities to formal health care providers requires much more than having close links in the community.

Theoretically, it also means that if communities are not prepared to receive and absorb the information and resources that come from outside the community, then instead of supporting the local systems, the inflow of these resources undermines local systems. The implementation of the VHT strategy was meant to harness these resources within the formal health care institutions by creating access routes to networks of power and influence [29]. Literature on community development shows that whereas linking social capital is critical for generating resources that are beneficial to the community, in most cases, communities may not have enough structures to absorb these resources [31]. Implementation of VHTs ought to have learnt from such examples.

### Power relations and VHTs failure to link communities to formal health care providers

Linking social capital postulates that linking individuals and structures across vertical relationships allows for the effective harnessing of resources that accrue once communities are connected to formal institutions of power and authority [31, 42, 54–56]. This depicts a relationship defined by how much control an entity has over resources. By connecting communities to formal health care facilities through CHWs, it was assumed that it promotes community interests in formal institutions and therefore develops the capacity to engage power structures and formal authorities ([31], p. 2207). Specifically, the implementation of the VHT strategy assumed that it would make the community demand utilisation of services from formal health care providers ([11] p.19).

Studies of CHWs programs hardly articulate how the interests of various stakeholders—community, CHWs, and formal health care providers—can be addressed. The introduction of the VHT strategy overlooked community perceptions and the power of formal health care professionals, particularly the attitudes of health care providers in facilitating the capacity of the VHTs to link community members with formal health care workers. This largely left the VHTs dependent on the formal health care providers for executing the referrals. In Senegal, Mladovsky et al. found that the dependency of community-based health insurance (CBHI) schemes on hospitals left the CBHI schemes with little negotiating power for their goals and interest [57]. In countries like Pakistan, the Lady Health Worker programme was hailed as effective partly because the programme invested in, among other things, maintaining strong ties with existing community resources such as traditional birth attendants [5]. Discussions with formal health care providers suggest that while they appreciated the role of VHTs as community-based workers, they were concerned about their (VHTs) demand for preferential treatment for the patients they refer at public health facilities. This attitude affected the nourishment of respectful and trusting ties between the communities, the VHTs and formal health care providers leading to what Putnam calls “unresponsive networks”[58, 59]. In hindsight, this presupposes a well-documented challenge to the successful implementation of CHW programmes, which is about the fear professional health workers have of losing their professional autonomy to non-professionals [5, 16, 60]. Yet, even when the literature discusses the fear of CHWs taking over the power of the professional health workers, the VHTs did not seem to possess the same power at community level as professional health workers did in the formal health care system. Their (VHTs) power at community level was over time eroded by failure to meet community expectations and the perceptions that VHTs were working for professional health workers. While our earlier observations revealed that a poorly executed selection process “saw the power of the community to select their own representatives usurped by their local leaders” [26], the VHTs appeared to have lost this power because of failure to meet community expectations as the VHT program progressed. But the fact that many VHTs were also political leaders at village level contrary to the VHT strategy provisions [11], we can adduce that political power was at play. Even then, this does not seem to have necessarily benefited the politicians as the senior medical officer had suggested about the “politics of control”. Given that we also did not necessarily witness where the VHTs were used for political mobilisation, we can argue that political manoeuvring affected the process but it did not empower the VHTs politically as they remain vulnerable to the professional elite. Instead, the VHTs no longer took initiative in referring people to the health centres or even following up cases such as TB or HIV in the community, which also increased their level of powerlessness in the eyes of the community members. If the key function of linking is to leverage critical resources including information and ideas from powerful institutions outside the communities [61] such as health care providers, then the introduction of VHTs did little to ensure that this leverage is achieved.

### Unmet community expectations and trust in formal health care providers

The unmet expectations affected the relationship between the community, the formal health care workers and the VHTs, which resulted into suspicion, accusations and counter-accusations. Community members “accused” the VHTs of working for the “other” and drawing salaries from the government; they also accused the government of failing to facilitate the work of the VHTs. The formal health care workers accused the VHTs of asking for privileges while faulting the government for attempting to use VHTs as a ploy to control people. On the other hand, the VHTs blamed the formal health care workers for failing to support them and accused the community of failing to appreciate their efforts and the government of abandoning them. Some of these accusations also emanated from what we have called *a transition failure* from AMREF to the formal health care system. It appears to have created a ‘counter-accusation effect’, confusion and subsequently undermined the perceived linkages.

While studies show that financial incentives are needed to motivate the CHWs, and that the willingness to work without pay reduces [62], we find that the practice of giving incentives needs to be sustainable. When incentives are not sustainable, as we have seen in the case of VHTs, it partly affects the motivation to do work and increasingly become alienated from the community members they serve. The literature that discusses CHWs motivations, attrition and remuneration appears to have concentrated on the impact of incentives on the motivation of the CHWs. However, this study also shows that attention needs to paid to how the incentives given to the CHWs can affect the trust of community members over time. Mladovsky et al., for example, show that ‘volunteering by CBHI staff built trust in CBHI among the target population.’ ([57] p. 776). While the VHTs were recruited as volunteers, some community members perceived them as drawing salaries from the government, and in practice, their work is facilitated leading to a perception that they occupy a privileged position. This means that the program should have invested in making the community members understand that the VHTs were community volunteers. However noble an intervention, it is only as good as understood and appreciated by the local people it is meant to serve.

From a theoretical stand point, linking social capital opines that effective linkages are based on ‘partnerships between the health care providers or public health entities and underserved communities’ ([33], p.7). According to Derose et al., such partnerships are a precursor to the protection against poor services and a form of accountability for the service recipients [63]. However, this also means that linkages and partnerships thrive on trust between health care providers and the marginalised communities [33]. The observed failure of the VHTs to deliver on their expectations affected the trust, enthusiasm and the relationship between the community and the VHTs. 

At the same time, the failure to recognise simple gestures such as preferential treatment for patients referred by VHTs affected the relationship between health workers and VHTs. In such an environment, it was difficult to engender a ‘system model of collaboration’ upon which partnerships thrive ([29], p. 1078).

### VHTs are no panacea to a failing health care system

Whereas the potential exists, VHTs are no panacea to weak health care systems. Whereas CHWs are supposed to be a cheaper option that is nearer to the community, responsive to community needs and linking communities to formal health care, they cannot be successful in a generally dysfunctional health system. The challenges raised in this study reflect the whole problem of system failure in Uganda’s health care system where decisions are largely politically driven. The system is not working well, be it at community or health facility levels or at referral hospitals. Health workers questioned the government’s commitment to equipping VHTs when the formal health care facilities are already poorly facilitated. VHTs questioned the nature of support received under the arrangement. We found for example, that indeed, when faced with multiple health care systems, the VHTs position is challenged. But most importantly, when a VHT hits a deadlock, as happened in the case of Okello, i.e. if a community member does not take the VHT’s advice, there is hardly any support from the formal health care system or government to help the VHT overcome this challenge. It puts to question the political will and calls for creative ways in which CHWs can be supported to navigate the clash between community cultural understanding of illness and disease and the biomedical interpretation which has been documented elsewhere [64]. The story of Okello’s son essentially summarises the limitations faced by VHTs in serving as a link to the community members’ access to formal health care facilities and failings of government. As the government of Uganda prepares to institute HEWs, this might simply become another failed intervention if the basic health systems failures are not addressed to enable VHTs link with the formal health care system.

### Limitations

The results presented in this paper are part of the broader study which addressed several issues relating to community health resources. The issues concerning failures and successes of VHTs were not initially the main objective of this study. As a result, we were not able to follow up various other issues that are equally central to the VHTs. However, this is compensated to some extent by the ethnographic approach involving three researchers embedded in the community for an extended period, which enabled us to study the community much more holistically and explore issues that are hardly captured by other methods. Second, the results were derived from a single community in Luwero District. The experiences of VHTs in Uganda are such that wherever they have been set up, they have been supported by NGOs. Depending on the NGO’s strength and level of engagement, the outcomes relating to the functioning of VHTs differ. Therefore, this might affect the generalizability of our results. However, we feel that the results raise some critical points which are relevant in other parts of the country where similar conditions pertain. Indeed, most rural parts of the country share many socio-economic characteristics. Problems are shared across contexts and so feasible solutions may also be related. The findings provide a starting point to study the linkages between community and formal health care through VHTs.

## Conclusions

The creation of a structure such as VHTs between communities and formal health care simply juxtaposes community health with formal health care. It projects a community as an “object” that can simply be “wired” to where services are but is devoid of the partnership arrangement theoretically espoused under the linking social capital framework. The VHTs assumed an ambiguous position between the community and the formal health care system, but their work reflected an extension of the formal health care structure into the community curtailing mutual linkages with the community as an integral part of the process.

From a theoretical view, the failures of VHTs as linking agents are embedded within the power relations that partly draw from the hierarchical health care system that places the community at the bottom of the ladder contrary to the WHO recommendation of a hub and spoke model of primary health care delivery [10]. Linking social capital espouses that studying interventions like VHTs requires an understanding of the power relations among health systems stakeholders.

The results have policy relevance given that VHTs remain central to Uganda’s national health care delivery. One of the policy implications is that there is need to plan for better transition of interventions like VHTs from NGO to government run programs. Better planning for transition would mean greater sustainability of NGO-initiated linkages and increased access to formal health care for communities. As linking social capital framework shows, for VHTs to effectively act as links between the community and formal health care and harness the resources that exist in institutions beyond the community, it is important to take into account the power relationships embedded in vertical relationships and forge a partnership between public health providers and the communities they serve.

## Abbreviations

AMREF: African Medical Research Fund
CHW(s): Community health worker(s)
CoHeRe: Developing Sustainable Community Health Resources
FGD: Focus group discussions
HC: Health Centre
HUMC: Health Unit Management Committee
VHT(s): Village health team(s)
